# Principle Research on a Single Mass Piezoelectric Six-Degrees-of-Freedom Accelerometer

**DOI:** 10.3390/s130810844

**Published:** 2013-08-16

**Authors:** Jun Liu, Min Li, Lan Qin, Jingcheng Liu

**Affiliations:** 1 Key Laboratory of Optoelectronics Technology and Systems Ministry of Education, Chongqing University, Chongqing 400044, China; E-Mails: limin780815@cqu.edu.cn (M.L.); qinlan@cqu.edu.cn (L.Q.); jcliu@cqu.edu.cn (J.L.); 2 College of Optoelectronic Engineering, Chongqing University, Chongqing 400044, China

**Keywords:** six-DOF accelerometer, piezoelectric sensor, finite element

## Abstract

A signal mass piezoelectric six-degrees-of-freedom (six-DOF) accelerometer is put forward in response to the need for health monitoring of the dynamic vibration characteristics of high grade digitally controlled machine tools. The operating principle of the piezoelectric six-degrees-of-freedom accelerometer is analyzed, and its structure model is constructed. The numerical simulation model (finite element model) of the six axis accelerometer is established. Piezoelectric quartz is chosen for the acceleration sensing element and conversion element, and its static sensitivity, static coupling interference and dynamic natural frequency, dynamic cross coupling are analyzed by ANSYS software. Research results show that the piezoelectric six-DOF accelerometer has advantages of simple and rational structure, correct sensing principle and mathematic model, good linearity, high rigidity, and theoretical natural frequency is more than 25 kHz, no nonlinear cross coupling and no complex decoupling work.

## 1. Introduction

Many motion controlled and measured systems, such as vehicles, automatic navigation, earthquake prediction devices, robot control systems, health monitoring of machine tools and other areas require determination of spatial vibration characteristics. This spatial vibration information can be described by the six-DOF acceleration motion. Six-axis accelerometers which can perform the simultaneous measurement of the six spatial components (three translational and three rotational) of acceleration are used as important sensing elements for detecting spatial vibration information.

At present, six-DOF acceleration sensing methods can be classified into the single-mass-spring-damper six-axis acceleration sensing approach (SPMSD approach) and the integrated six-axis acceleration sensing approach based on multiple single-axis accelerometers (MSAAs approach) [1]. The SPMSD approach was mainly used to develop six-axis accelerometers, that sense the six-DOF acceleration by accessing the displacement of an inertial mass relative to the rigid body or the deformations of the elastic elements of the spring—dampers connecting the inertial mass with the rigid body. The MSAAs approach was explored in the research of gyro-free strapdown inertial navigation systems and six-DOF acceleration measurement systems, and senses the six-DOF acceleration by accessing and decoupling the outputs from the MSAAs positioned and oriented on a rigid body according to appropriate configurations.

According to the type of conversion element used, the six-axis accelerometers based on the SPMSD approach can be classified into elastic style [2], electrically suspension style [3], superconducting suspension style [4], spring photoelectric style and so on [5]. These sensors have advantages of compact conformation and high degree of integration, however, they have the disadvantages of complicated production process, high cost, narrow scope of application, difficult miniaturization and so on, meanwhile, due to the fact the SPMSD approach is similar to the research idea of elastic style six-axis force/torque sensors, there are two bottleneck contradictions, including the degree of complexity of the structures and the difficulty of decoupling, high stiffness and high sensitivity [6].

According to the number of single-axis accelerometers used, six-DOF accelerometers based on the MSAAs approach can be classified into six-single-axis accelerometer style and nine-single-axis accelerometer style [7]. These six-axis accelerometers have the advantages of a simple research idea, simple production process and so on, however, they have the disadvantages of high cost, complicated structure, and large volume. They are also difficult to install and realize structural miniaturization.

In order to overcome the shortcomings of the six axis accelerometer based on the SPMSD and MSAAs approaches, in this paper, a novel six-DOF accelerometer sensing principle based on a single inertial mass is presented. Piezoelectric quartz is chosen for the force sensing element and conversion element. The operating principle of the proposed six-axis accelerometer is analyzed, a structure model is built, a numerical finite element model of the piezoelectric six-DOF accelerometer is set up, and its static sensitivity, static coupling interference and dynamic natural frequency, and dynamic cross coupling are analyzed by ANSYS software. The research results show that the measurement principle of the piezoelectric six-DOF accelerometer is correct.

## 2. Measurement Principle and Structure Model

**Figure 1.**
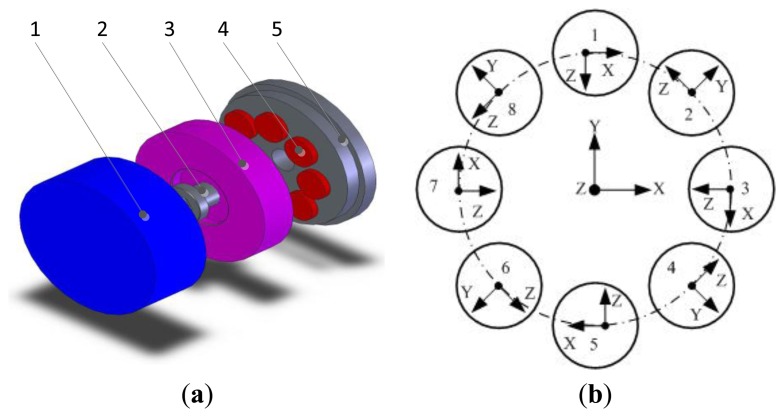
Schematic diagram of the 6-axis accelerometer. (**a**) Exploded view of the six-DOF accelerometer; (**b**) Spacial layout structure schematic of the quartz chip groups.

The mechanical structure of the piezoelectric six-DOF accelerometer is shown in Figure 1a. This piezoelectric six-DOF accelerometer belong to the disaggregated structure piezoelectric sensor type. Piezoelectric quartz is chosen for the sensing element and conversion element of the six-DOF accelerometer. The piezoelectric six-DOF accelerometer is composed of a cover (1), pretension bolts (2), cylindrical inertial mass (3), piezoelectric quartz crystal chip groups (4), pedestal (5), signal output electrodes, insulating filler and other major parts. The quartz crystal groups are clamped between the inertial mass and the pedestal, and are symmetrically distributed on the upper surface of the quartz crystal chip group's mounting boss. To improve the impedance characteristics and anti-jamming performance of the piezoelectric six-axis accelerometer, the piezoelectric quartz crystal chip groups are designed for a double-layer structure.

### 2.1. Measurement Principle

The spatial layout structure schematic of the quartz chip groups within the piezoelectric six-axis accelerometer is shown in Figure 1b. Assuming that the measured linear acceleration and angular acceleration are *a_x_*, *a_y_*, *a_z_*, *am_x_*, *am_y_* and *am_z_*, the piezoelectric quartz crystal chip groups response outputs are *A_X_*, *A_Y_*, *A_Z_*, *AM_X_*, *AM_Y_* and *AM_Z_*. Eight quartz crystal chip groups are evenly distributed on the same circle. Four groups of Y0^0^-crystals are distributed on the nodes of X and Y axes and the quartz crystal chip groups distribution circle, and are used for the measurement of *a_x_*, *a_y_* and *am_z_*. Four groups of X0^0^-crystals are distributed on other locations and are used for the measurement of *a_z_*, *am_x_*, and *am_y_*. Each group quartz crystal chip group corresponds to a one-channel output signal and can obtain 6-channel signals via pretreatment of 8-channel signals, and the six-axis acceleration can be measured via operation of a decoupling matrix. Equation (1) represents the 8-channel signal to 6-channel signal conversion expression in which the subscript letters represent spatial axes and the value index indicates the number of quartz chip groups, F represent the inertial force that generated by acceleration:

(1) $$\{\begin{array}{l} A_{X}=k_{a_{x}}(F_{Y1}-F_{Y5})\\ A_{Y}=k_{a_{y}}(F_{Y3}-F_{Y7})\\ A_{Z}=k_{a_{z}}(F_{Z2}+F_{Z4}+F_{Z6}+F_{Z8})\\ AM_{X}=k_{am_{x}}[(F_{Z2}+F_{Z8})-(F_{Z4}+F_{Z6})]\\ AM_{Y}=k_{am_{y}}[(F_{Z6}+F_{Z8})-(F_{Z2}+F_{Z4})]\\ AM_{Z}=k_{am_{z}}(F_{X1}+F_{X3}+F_{X5}+F_{X7}) \end{array}(1)$$

Due to the influence of the piezoelectric six-axis accelerometer structure, the layout of the quartz chip group, the quantity and production level (among other factors), the arrangement of the quantity of quartz crystal cells and the production level, the actual conditions do not fully meet the above assumption in practice. Therefore, the acceleration transfer coefficients of *k_a_x__*_,_*k_a_y__*_,_*k_a_z__*_,_*k_am_x__*_,_*k_am_y__*_and_*k_am_z__* were introduced into this study, and the acceleration coefficient calculation study will be shown in the subsequent papers.

### 2.2. Structure Model

To simplify the analysis, the following assumptions are adopted: the rigidities of the quartz crystal chip groups are identical, with equal sensitivity and symmetry uniform distribution. The inertial mass is a rigid body with the same stiffness in all directions, equal sensitivity, and uniform distribution. The directions of *a_z_*, *am_x_* and *am_y_* are distributed according to the lever principle on quartz crystal chips, and *a_x_*, *a_y_* and *am_z_* are evenly distributed.

**Figure 2.**
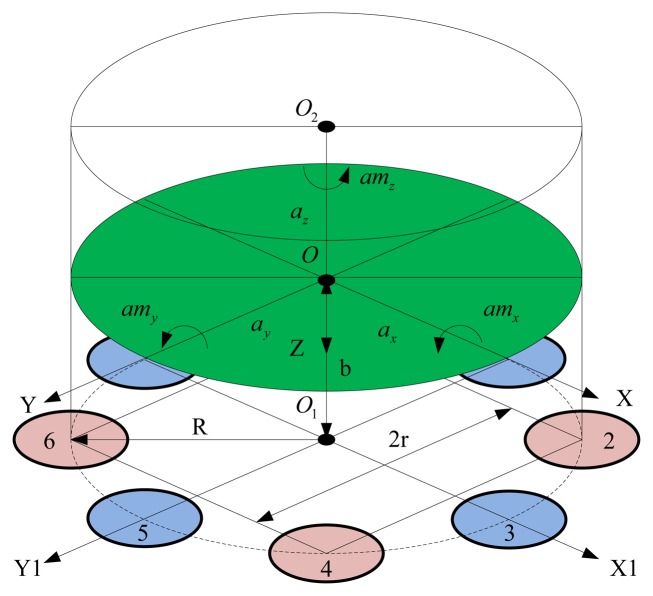
Block diagram of the sensor's structure.

Figure 2 shows the block diagram of the piezoelectric six-axis accelerometer structure. The designation O-XYZ represents the coordinate system of centroid of inertial mass and O_1_-X_1_Y_1_Z_1_ denotes the installation layout position coordinate system of quartz crystal chip groups. The quartz crystal chip groups are arranged along the same circle with radius is R, the distance between the quartz chip group 2 and 4 is r = Rcos45°, and the distance between the centroid of inertial mass and the surface of quartz crystal chip groups is b, the inertial force that generated by acceleration acting on each quartz crystal chip groups can be expressed by Equation (2):

(2) $$\{\begin{array}{l} F_{X1}=a_{x}/8+am_{z}/8R+am_{y}/R\\ F_{X5}=-a_{x}/8+am_{z}/8R-am_{y}/R\\ F_{Y3}=a_{y}/8+am_{z}/8R-am_{x}/R\\ F_{Y7}=-a_{y}/8+am_{z}/8R+am_{x}/R\\ F_{Z2}=a_{z}/8+[-a_{y}b-a_{x}b+am_{x}-am_{y}]/3r\\ F_{Z4}=a_{z}/8+[a_{y}b-a_{x}b-am_{x}-am_{y}]/3r\\ F_{Z6}=a_{z}/8+[a_{y}b+a_{x}b-am_{x}+am_{y}]/3r\\ F_{Z8}=a_{z}/8+[-a_{y}b+a_{x}b+am_{x}+am_{y}]/3r \end{array}$$

According to Equations (1) and (2), we can obtain the following relational expression of the six-DOF accelerometer's structural model:

(3) $$\{\begin{array}{l} A_{X}=(a_{x}/4+2am_{y}/R)k_{a_{x}}\\ A_{Y}=(a_{y}/4+2am_{x}/R)k_{a_{y}}\\ A_{Z}=(a_{z}/2)k_{a_{z}}\\ AM_{X}=(4am_{x}/3r-4a_{y}b/3r)k_{am_{x}}\\ AM_{Y}=(4am_{y}/3r+4a_{x}b/3r)k_{am_{y}}\\ AM_{Z}=(am_{z}/2R)k_{am_{z}} \end{array}$$

As can be seen from Equation (3), due to the influence of the sensor structure, the cross coupling interferences of six-DOF accelerometer take place in the *a_y_*, *am_x_*, *a_x_*, *am_y_* directions.These interferences are different from traditional nonlinear coupling, and can be eliminated using a mathematical compensation method.

## 3. Experiment of Numerical Simulation

To verify the effectiveness of the piezoelectric six-axis accelerometer structure model, ANSYS software is used to pre-assess the piezoelectric six-DOF accelerometer's static and dynamic characteristics. ANSYS software has powerful analysis capability in the coupled field, which is the preferred software in the field of piezoelectric analysis. However, its physical model modeling function is weak, making it unsuitable for creating complex physical models. So the analysis process of the six-DOF accelerometer primarily applies a modeling approach and load application method. Table 1 lists the main structural dimensions of the sensor examined in this paper.

### 3.1. FEM Model

#### Modeling approach

In the first step, the physical structural model of the piezoelectric six-axis accelerometer is built with CAD software (*i.e.*, SolidWorks, PRE/E), and the physical structural model is imported into the ANSYS software. In the simulation process, coupling unit SOLID98 was chosen as the element type of the quartz crystal, and the piezoelectric coefficient matrix, the elastic coefficient matrix and the dielectric matrix can be defined according to the characteristics of the piezoelectric quartz crystal. Unit SOLID95 was chosen as the element type of the other components of the six-axis accelerometer, and stainless steel was chosen as the material of the six-axis accelerometer's other components. The piezoelectric six-axis accelerometer's work coordinates and quartz crystal chip groups' local coordinates can be constructed according to the operating conditions of the sensor and digestion type of the quartz crystal chips. Finally, according to the actual computational requirements, the meshing method is determined to complete the meshing of the piezoelectric six-DOF accelerometer.

#### Load application method

This approach includes the installation constraints and acceleration loading on the piezoelectric six-axis accelerometer. The constraints set adhere to the sensor's installation status and the preload force is applied through a section of the inertial mass. The degree of freedom of the pedestal's mounting surface is zero. The measured accelerations are applied to the key point, which is established on the Z axis and located in the same plane as the upper surface of the inertial mass. Additionally, the key point and the upper surface of the inertial mass are built in the rigid region.

### 3.2. Static Characteristics Simulation Analysis

**Figure 3.**
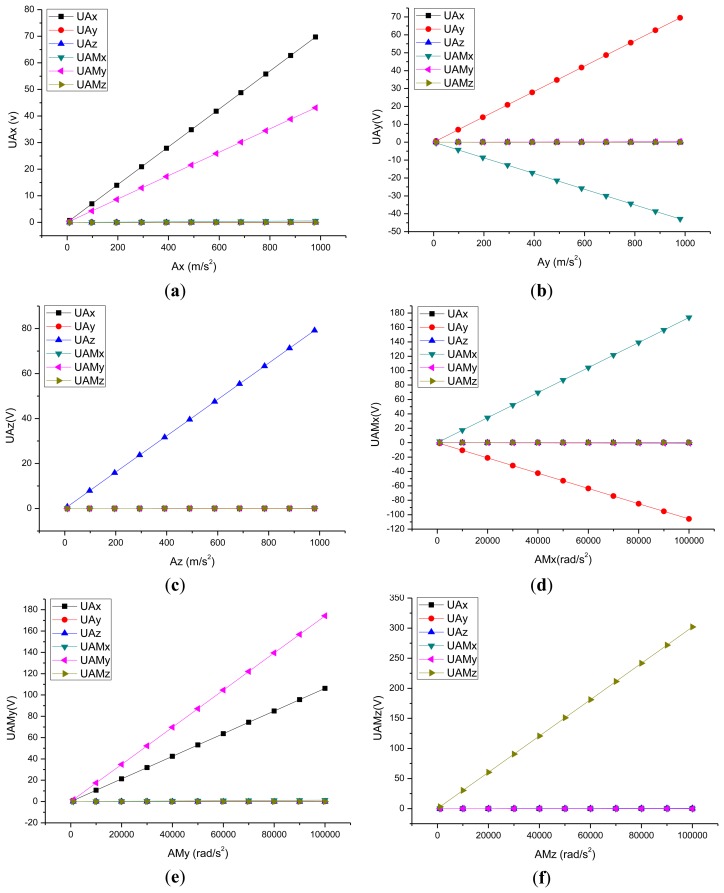
Piezoelectric six-DOF sensor's input acceleration and output voltage curve. (**a**) X-axis linear acceleration; (**b**) Y-axis linear acceleration; (**c**) Z-axis linear acceleration; (**d**) X-axis angular acceleration; (**e**) Y-axis angular acceleration; (**f**) Z-axis angular acceleration.

In the piezoelectric coupled field process based on the ANSYS software, the potential difference between the surface of piezoelectric quartz crystal chip can be obtained, so in the paper, the relationship between the potential difference and the acceleration was selected instead of the relationship between the acceleration input and the potential output. Figure 3 show the curves of the input acceleration and the output potential in the directions of *a_x_*, *a_y_*, *a_z_*, *am_x_*, *am_y_* and *am_z_*. The symbol U represents the potential. According to these curves, it can get the six-DOF accelerometer's C matrix as shown in Equation (4):

(4) $$C_{U}=\left[\begin{array}{llllll} 6.970E-01 & 1.070E-03 & -4.00E-04 & 5.600E-03 & 4.310E-01 & -5.10E-04\\ -2.40E-04 & 6.950E-01 & 1.800E-04 & -4.29E-01 & 2.880E-03 & -5.00E-04\\ -1.00E-05 & -7.00E-05 & -7.92E-01 & 9.800E-04 & 7.600E-04 & 8.900E-04\\ 0.000E+00 & -1.06E-03 & -2.00E-06 & 1.740E-03 & -9.00E-06 & 1.000E-06\\ 1.060E-03 & 2.000E-06 & -1.00E-06 & 1.400E-05 & 1.740E-03 & -1.00E-06\\ 2.000E-06 & 2.000E-06 & 5.000E-06 & -1.00E-06 & -1.00E-06 & -3.02E-03 \end{array}\right]$$

Table 2 shows the static sensitivity and cross coupling characteristics of the piezoelectric six-DOF accelerometer, which was derived from Figure 3 and Equations (4). It can be seen that the input load is linear with output potential, the static sensitivity is 0.697 V/g in the *a_x_* direction, 0.695 V/g in the *a_y_* direction, 0.792 V/g in the *a_z_* direction, 0.00174 V/(rad/s^2^) in the *am_x_* direction, 0.00174 V/(rad/s^2^) in the *am_y_* direction, 0.00302 V/(rad/s^2^) in the *am_z_* direction. The cross coupling interferences of the piezoelectric six-axis accelerometer only take place in *a_y_*, *am_x_*, *a_x_*, *am_y_* directions, and these interferences are the linear coupling interferences, which are different from traditional nonlinear coupling and can be eliminated using a mathematical compensation method (the compensation method can be obtained via our other paper about the calculation of six axis force sensor [8]), and these results were consistent with the results based on the structural model of the six-axis accelerometer.

### 3.3. Dynamic Characteristics Simulation Analysis

**Figure 4.**
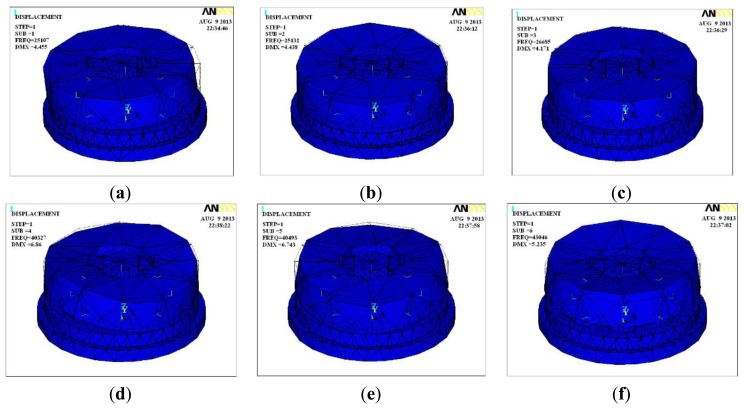
Inherent vibration modes of the piezoelectric six-DOF accelerometer. (**a**) 1th vibration mode; (**b**) 2th vibration mode; (**c**) 3th vibration mode; (**d**) 4th vibration mode; (**e**) 5th vibration mode; (**f**) 6th vibration mode.

Research on the piezoelectric six-DOF accelerometer's dynamic characteristics involves the study of its natural frequency. Modal analysis, harmonic analysis and other methods based on ANSYS software can be used to estimate the natural frequency of the six-axis accelerometer. Figure 4 shows the natural frequency of first six ranks and its vibration modes for the six-DOF accelerometer, Table 3 shows the modes traits of the first six ranks. Since the structure of six-axis acceleration sensor belongs to a symmetrical structure type, the natural frequency of the inertial force in the direction X and Y, the inertial torque in the direction Z are the same, the minimum theoretical value of six-axis acceleration sensor's natural frequency can reach to 25 kHz, however, the six-DOF accelerometer's amplitude frequency characteristics of each frequency point can't be obtained with the modal analysis method.

**Figure 5.**
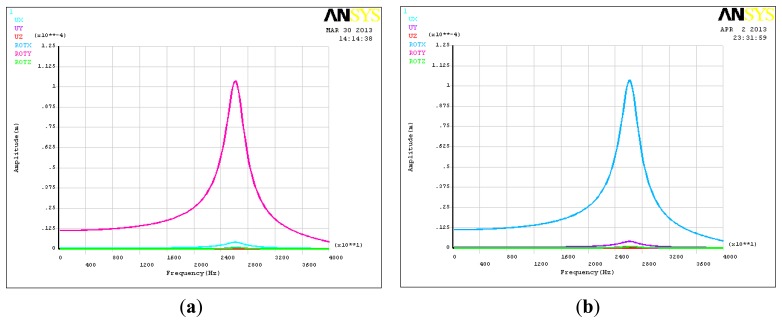

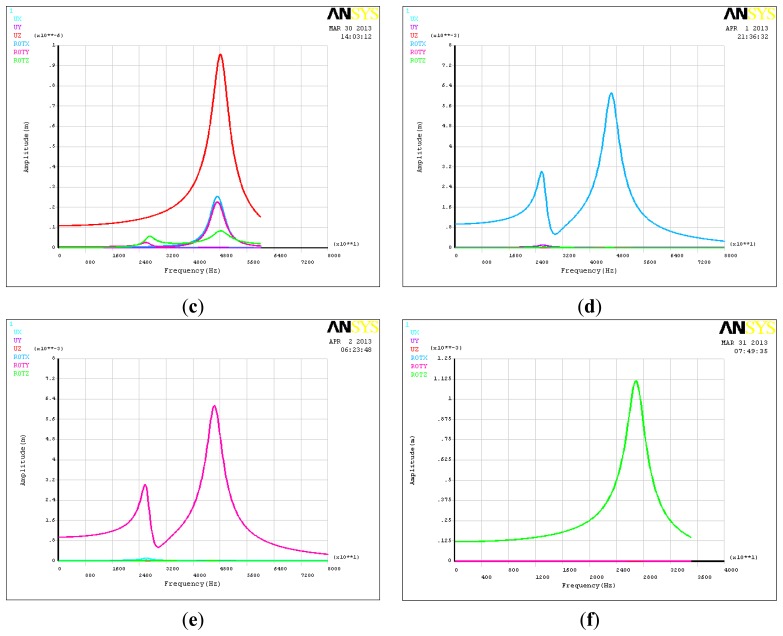
The amplitude-frequency response curve of piezoelectric six-DOF accelerometer. (**a**) X-axis linear acceleration; (**b**) Y-axis linear acceleration; (**c**) Z-axis linear acceleration. (**d**) X-axis angular acceleration; (**e**) Y-axis angular acceleration; (**f**) Z-axis angular acceleration.

To study the amplitude frequency characteristics of the piezoelectric six-DOF accelerometer, the harmonic analysis method was chosen. Figure 5 shows the amplitude frequency characteristics curves of six-DOF accelerometer. As can be seen that the linear acceleration and the angular acceleration of the X,Y,Z directions are 26.2 kHz, 26.2 kHz, 48.0 kHz, 47.2 kHz, 46.4 kHz and 26.5 kHz, and there is a resonance point at 25.8 kHz in the angular acceleration of X and Y direction. Table 4 shows the dynamic cross coupling characteristics of the six axis acceleration sensor, which take place at the frequency point of 18.6 kHz. This frequency point is the 0.707 times lowest resonant frequency of the six-DOF accelerometer. It is different between the dynamic cross coupling and static cross coupling, there are some nonlinear components in the dynamic cross coupling characteristic, and as the test frequency declines, the dynamic cross coupling of the six DOF accelerometer will drop slowly.

### 3.4. Simulation Analysis of Combined Loading

**Figure 6.**
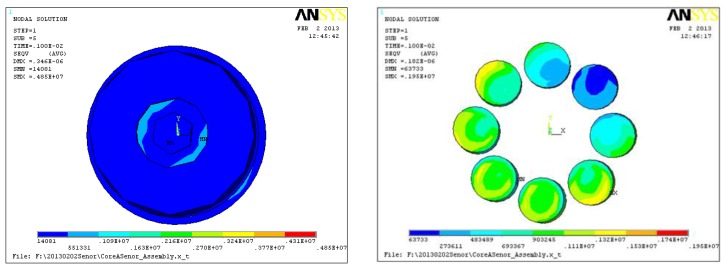
The strain cloud of six-DOF accelerometer's core parts and quartz crystal chip groups under the composite loads.

Figure 6 shows the strain cloud of the six-DOF accelerometer's core parts, Table 5 shows the input and output decoupling results, on the condition that the composite loads acted on the six-DOF accelerometer. It can be seen that these static cross coupling interferences are different from traditional nonlinear coupling and can be eliminated using a mathematical compensation method. Due to the cross coupling interferences of six-axis accelerometer take place in the *a_y_*, *am_x_*, *a_x_*, *am_y_* directions, so in these directions there are some relatively large calculation errors.

## 4. Conclusions

In this paper, the feasibility of the six-DOF acceleration sensing principle based on the SPMSD approach has been explored. On this basis, the principle and characteristics of the six-DOF acceleration sensing method based on signal mass piezoelectric six axis accelerometer has been proposed and analyzed. According to the research results, the following conclusions can be obtained:

1. The six-axis acceleration sensing principle and the structure of the single inertial mass piezoelectric six-DOF accelerometer can effectively provide the 6-axis acceleration information.
2. Due to the influence of the six-DOF accelerometer's spatial structure, there are some cross coupling interferences of six-axis accelerometer take place in the *a_y_*, *am_x_*, *a_x_*, *am_y_* directions, and these static cross coupling interferences are different from traditional nonlinear coupling and can be eliminated using a mathematical compensation method.
3. According to the one-dimensional miniature piezoelectric accelerometer research approach of the literature [9], if this six-axis acceleration research approach were to be combined with MEMS technology, miniaturization of the single inertial mass piezoelectric six-DOF accelerometer is expected to be possible.
4. Compared with commercial single or multi-axis linear accelerometers or angular accelerometers, although the sensitivity of piezoelectric six axis accelerometer is slightly lower, its natural frequency is expected to increase 2–5 times.

## Figures and Tables

**Table 1.** Main structural parameters of the six-DOF accelerometer model.

**Component**	**Thickness (mm)**	**Outside Diameter (mm)**	**Material**	**Elastic Modulus (Pa)**	**Density (Kg/m^3^)**
Inertial mass	10	46	1Cr18Ni9Ti	2.1e11	7,900
Mounting boss	4	46	1Cr18Ni9Ti	2.1e11	7,900
Mounting pedestal	4	50	1Cr18Ni9Ti	2.1e11	7,900
Quartz crystal chip	2	10	SiO_2_	8.0e10	2,650

**Table 2.** Simulation results of static sensitivity and cross coupling.

**Load**	**Static Sensitivity and Cross Coupling**					

	**Ax**	**Ay**	**Az**	**AMx**	**AMy**	**AMz**
Ax(V/g)	0.697	0.15%	−0.06%	0.80%	61.84%	−0.07%
Ay(V/g)	−0.03%	0.695	0.03%	−61.78%	0.41%	−0.07%
Az(V/g)	0.00%	−0.01%	0.792	0.12%	0.10%	0.11%
AMx(V/(rad/s^2^))	0.00%	−60.93%	v0.12%	0.00174	−0.52%	0.06%
AMy(V/(rad/s^2^))	60.93%	0.11%	−0.06%	0.80%	0.00174	−0.06%
AMz(V/(rad/s^2^))	0.07%	0.07%	0.17%	−0.03%	−0.03%	0.00302

**Table 3.** Simulation results of modal analysis.

**Rank**	**Natural Frequency** (**Hz**)	**VIBRATION Mode**
1	25,107	The linear vibration of inertial mass along the X-axis
2	25,132	The linear vibration of inertial mass along the Y-axis
3	26,685	The rotational vibration of inertial mass along the Z-axis
4	40,327	The folded vibration of inertial mass along the bisector of X and Y-axis
5	40,493	The folded vibration of inertial mass along the Y-axis
6	43,046	The linear vibration of the outer edge of inertial mass along the Z-axis

**Table 5.** Analytical results of composite loads.

**Load Category**	**Input Value**	**Output Potential Difference**	**Output of Decoupling**	**Relative Error**
Ax	490 m/s^2^	133.111 V	488.70 m/s^2^	0.27%
Ay	490 m/s^2^	−62.731 V	491.09 m/s^2^	−0.22%
Az	490 m/s^2^	−39.512 V	489.48 m/s^2^	0.11%
AMx	92,395 rad/s^2^	140.746 V	92,464.11 rad/s^2^	−0.07%
AMy	92,395 rad/s^2^	182.008 V	92,501.62 rad/s^2^	−0.12%
AMz	65,333 rad/s^2^	−197.244 V	65,332.21 rad/s^2^	0.00%
